# Physical Comorbidity According to Diagnoses and Sex among Psychiatric Inpatients in South Korea

**DOI:** 10.3390/ijerph18084187

**Published:** 2021-04-15

**Authors:** Suin Park, Go-Un Kim, Hyunlye Kim

**Affiliations:** 1 College of Nursing, Kosin University, Busan 49267, Korea; suinpark@kosin.ac.kr; 2 College of Nursing, Yonsei University, Seoul 03722, Korea; 3 Department of Nursing, College of Medicine, Chosun University, Gwangju 61452, Korea; hlkim5207@chosun.ac.kr

**Keywords:** mental disorders, sex, physical comorbidity

## Abstract

People with mental disorders are susceptible to physical comorbidities. Mind–body interventions are important for improving health outcomes. We examined the prevalence of physical comorbidities and their differences by diagnoses and sex among psychiatric inpatients. The dataset, from National Health Insurance claims data, included 48,902 adult inpatients admitted to psychiatric wards for at least 2 days in 2016 treated for schizophrenia, schizotypal and delusional disorders, or mood disorders. We identified 26 physical comorbidities using the Elixhauser comorbidity measure. Among schizophrenia-related disorders, other neurological disorders were most common, then liver disease and chronic pulmonary disease. Among mood disorders, liver disease was most common, then uncomplicated hypertension and chronic pulmonary disease. Most comorbid physical diseases (except other neurological disorders) were more prevalent in mood disorders than schizophrenia-related disorders. Male and female patients with schizophrenia-related disorders showed similar comorbidity prevalence patterns by sex. Among patients with mood disorders, liver disease was most prevalent in males and third-most in females. In both diagnostic groups, liver disease and uncomplicated diabetes mellitus were more prevalent in males, and hypothyroidism in females. Mental health professionals should refer to a specialist to manage physical diseases via early assessments and optimal interventions for physical comorbidities in psychiatric patients.

## 1. Introduction

Mind–body interaction, or the concept that the mind and body are connected, is useful for understanding psychophysiology in the field of mental health [1]. For instance, strong evidence has accumulated about the link between hypothalamic–pituitary–adrenal (HPA) axis dysregulation and the risk of developing mental illness [2]. In addition, many systemic disorders associated with a higher incidence of mental illness seem to involve a significant inflammatory component [2]. Demographic changes and medical advancements over the past decades have significantly increased the comorbidity of mental and physical illnesses, both of which are expanding across all ages, and this growth trend will continue [3]. Mental health professionals accept that all diseases have a mental and physical component; thus, people with severe mental illness (SMI) should be approached holistically. 

The comorbidity of mental and physical illnesses worsens the prognosis of both types of disorder, significantly increases the cost of treatment, and increases mortality [3]. Coexisting physical and mental disorders can worsen the course of illness, leading to hospital readmission due to non-psychiatric and psychiatric reasons [4]. A 12-year follow-up study of patients with schizophrenia admitted to a general hospital reported a higher rate of emergency admissions, longer average hospital stay, higher number of hospital admissions, reduced survival time, and a nearly two-fold increase in mortality rate compared with the control population [5]. Depression is associated with an approximately 50% increase in medical costs due to chronic medical illnesses, even after controlling for the severity of the physical illness [6]. In a study using National Health Insurance (NHI) claims data in South Korea, the number of chronic physical diseases in patients with schizophrenia was identified as a factor affecting medical costs [7].

A retrospective cohort study using the Clinical Practice Research Datalink, the UK’s primary care database, found that the annual prevalence rates of all physical comorbidities were higher in those with SMI than in those without [8]. A review study using broad searches from MEDLINE (1966–2010) revealed that nutritional and metabolic diseases, cardiovascular diseases, viral diseases, respiratory tract diseases, musculoskeletal diseases, sexual dysfunction, pregnancy complications, stomatognathic diseases, and possibly obesity-related cancers were more prevalent among people with SMI than in the general population [9]. 

Physical comorbidities in SMI are often associated with the side effects of psychotropic agents and unhealthy lifestyle factors [9]. Antipsychotic agents are particularly likely to increase the risks of obesity, metabolic syndrome, sedation, sexual dysfunction, postural hypotension, arrhythmia, and heart attack [10]. Health habits related to comorbid diseases in patients with SMI include high smoking rates, poor diet, lack of exercise, and unsafe sexual behaviors [11]. However, people with SMI often find it difficult to control their health-related behaviors because of psycho–behavioral symptoms, and psychopharmacological treatment cannot be avoided. 

Previous studies have been conducted on physical comorbidities according to the type of mental disorder including schizophrenia [5,12,13,14], psychosis [15], mood disorder [16], and bipolar disorder [17,18]. Regarding sex-related differences, major depressive disorder was associated with cardiometabolic comorbidity and women were at a higher risk than men of being afflicted by it [19]. In Korea, Lee et al. reported the prevalence of chronic physical comorbidities in patients with schizophrenia only [7]. However, there are insufficient studies on the differences in the physical comorbidities according to diagnoses and sex in people with SMI in Korea. To study the factors involved in various aspects of physical comorbidities, an approach that considers diagnosis and sex is necessary.

According to the recently published National Mental Health Statistics 2019 report in Korea, patients diagnosed with schizophrenia, schizotypal and delusional disorders (International Classification of Diseases, 10th revision (ICD-10): F2x; schizophrenia-related disorders), and mood disorders (ICD-10: F3x) accounted for approximately 67.9% of inpatients in mental hospitals [20]. Thus, these two classes of mental disorder are central to mental health care services in psychiatric wards. 

We assessed the current state of physical comorbidities in inpatients with schizophrenia-related disorders and mood disorders using the NHI claims data for South Korea. This study aimed to determine the prevalence of physical comorbidities and their differences according to diagnoses and sex among psychiatric patients. The findings of this study will help mental health professionals realize holistic care for people with SMI by providing basic data for exploring therapeutic approaches to their physical comorbidities. 

## 2. Materials and Methods 

### 2.1. Design and Data Source

This study is a secondary analysis of Korean NHI data aimed to determine the prevalence and characteristics of physical comorbidities in schizophrenia-related disorders and mood disorders. We used the 2015–2016 NHI claims data submitted to the Health Insurance Review and Assessment Service (HIRA) as well as part of a patient dataset constructed by Park et al. [21] based on HIRA data. This study was approved by the Institutional Review Board of Dongguk University (approval no.: DGU IRB 20190028-01; date: 18 October 2019) and the HIRA. In accordance with the procedures for big data analysis, we removed any information identifying the patients or medical institutions from the data.

### 2.2. Study Sample

The study cohort comprised 76,882 patients extracted from the dataset of Park et al. [21] using the same data source. Of these, we selected data from 48,902 patients (in 634 hospitals) whose primary diagnosis was schizophrenia-related disorders (F2x) or mood disorders (F3x), after excluding those with mental and behavioral disorders due to alcohol use. Our dataset contained information on patients aged 19 years or older who received inpatient treatment for 2 days or longer in the psychiatric department of tertiary or general hospitals, hospitals (intermediate in size to general hospitals and clinics in South Korea), psychiatric hospitals, or clinics from January 2016 to December 2016. The final dataset was constructed by merging the patient data with the Elixhauser comorbidity measure (ECM) scores calculated using the primary and subordinate diagnostic codes. The ECM index is a database-derived comorbidity measure that efficiently captures and discriminates comorbidities among large patient populations [22,23,24]. Further details on data selection and patient dataset construction are presented in Park et al.’s study [21].

### 2.3. Variables

General characteristics included age, sex, type of insurance, and previous psychiatric hospitalizations within the last year. To identify comorbid diseases that occurred within a year before admission to the psychiatric department in 2016, we used the diagnostic codes recorded in outpatient and inpatient care records in all medical departments from 2015 to 2016. The set of physical comorbidities comprised 26 physical diseases, excluding 4 diseases corresponding to mental disorders (alcohol abuse, drug abuse, psychoses, depression), among Elixhauser’s 30 comorbidities. The ECM score was calculated by assigning one point to each of the 26 physical comorbidities and then summing all points according to a method described previously [24]. We also followed the ICD-10 coding algorithm for defining Elixhauser’s comorbidity set by the ICD-9-CM coding system, which was developed by Quan et al. [25]. ECM demonstrated excellent external discrimination for in-hospital mortality of all-causes [26] and reasonable inter-rater agreement (kappa (κ) coefficient ≥ 0.4) for cardiorespiratory and oncological comorbidities [27].

### 2.4. Statistical Analyses

The data analysis was performed using SAS Enterprise Guide 6.1 (SAS ver. 9.4, windows version; SAS Institute Inc., Cary, NC, USA) software. Statistical analyses were based on two-sided tests at a significance level of 0.05. Descriptive statistics were used to confirm the general characteristics and the prevalence of comorbid physical diseases. Differences in general characteristics among the diagnostic groups were analyzed using the chi-square or independent *t*-tests. A chi-square test was performed for each of the 26 diseases to determine differences in the prevalence of comorbid physical diseases according to diagnoses and sex. 

## 3. Results

### 3.1. General Characteristics

Of the 48,902 subjects in 634 hospitals, 26,314 (53.8%) had schizophrenia-related disorders (schizophrenia, schizotypal and delusional disorders), and 22,588 (46.2%) had mood disorders. The overall mean age was 48.33 (±15.80) years; the mean age was higher in those with mood disorders compared with schizophrenia-related disorders. The schizophrenia-related disorders group included slightly more males (50.5%) than females, whereas the mood disorders group had more females (57.2%) than males (*χ*^2^ = 290.20, *p* < 0.001). Patients covered by health insurance constituted 75.6% of the mood disorders group, a higher proportion than in the schizophrenia-related disorders group (55.3%; *χ*^2^ = 2190.30, *p* < 0.001). The median length of stay was 28 (interquartile range (IQR) = 49) days, and schizophrenia-related disorders had longer length of stay (median = 34, IQR = 68) than mood disorders (median = 20, IQR = 30; *U* = 467,025,211.50, *p* > 0.001). 46.9% of patients with schizophrenia-related disorders were hospitalized in hospitals, followed by psychiatric hospitals (23.3%), and 35.1% of patients with mood disorders were hospitalized in hospitals, followed by general hospitals (29.0%; *χ*^2^ = 2497.02, *p* < 0.001). The rate of previous psychiatric hospitalizations within the last year was 45.5% in the schizophrenia-related disorders group versus 24.0% in the mood disorders group (*χ*^2^ = 2452.62, *p* < 0.001). The proportion of patients with an ECM score ≥ 1 during the last year was significantly higher in the mood disorders group than schizophrenia-related disorders group (76.4% vs. 68.6%; *χ*^2^ = 369.19, *p* < 0.001; Table 1).

### 3.2. Prevalence of Comorbid Physical Diseases According to Diagnoses

The top 10 most prevalent comorbid physical diseases are listed according to diagnoses in Table 2. In the schizophrenia-related disorders group, “other neurological disorders” were the most frequent comorbidity, followed by liver disease, chronic pulmonary disease, uncomplicated hypertension (HTN), and uncomplicated diabetes mellitus (DM). Among patients diagnosed with mood disorders, liver disease was the most common, followed by uncomplicated HTN, chronic pulmonary disease, other neurological disorders, and peptic ulcer disease excluding bleeding.

### 3.3. Differences in Comorbid Physical Diseases According to Diagnoses

Table 3 shows a comparison of comorbid physical disease prevalence according to diagnostic group. Among the 26 ECM diseases, only other neurological disorders showed a significantly higher prevalence in the schizophrenia-related disorders group compared with the mood disorders group (*χ*^2^ = 405.45, *p* < 0.001). The prevalence of all diseases, except obesity and human immunodeficiency virus infection/acquired immunodeficiency syndrome, was higher in the mood disorders than schizophrenia-related disorders. 

### 3.4. Prevalence of Comorbid Physical Diseases According to Diagnoses and Sex

Table 4 shows the top 10 most prevalent comorbid physical diseases according to sex for each diagnostic group. With regard to schizophrenia-related disorders, males and females exhibited the same comorbid disease ranking from 1 to 6. The highest ranking comorbidity was other neurological disorders (males 33.8%, females 30.8%), followed by liver disease (males 25.5%, females 21.1%), chronic pulmonary disease (males 19.7%, females 20.4%), uncomplicated HTN (males 19.7%, females 16.4%), uncomplicated DM (males 16.7%, females 14.8%), and peptic ulcer disease excluding bleeding (males 11.4%, females 12.1%). For those with mood disorders, the prevalence rankings differed between males and females. Among males, the five most prevalent diseases were liver disease (34.9%), uncomplicated HTN (28.9%), chronic pulmonary disease (24.9%), other neurological disorders (24.2%), and uncomplicated DM (22.0%); in females, the five most prevalent diseases were chronic pulmonary disease (31.1%), uncomplicated HTN (30.6%), liver disease (30.2%), peptic ulcer disease excluding bleeding (24.5%), and other neurological disorders (23.9%).

### 3.5. Differences in Comorbid Physical Disease Prevalence According to Diagnoses and Sex

Table 5 shows the differences in the prevalence of comorbid physical diseases according to diagnoses and sex. The list of comorbid physical diseases presented here includes the 10 diseases from Table 4 with the highest prevalence by diagnoses and sex. In the schizophrenia-related disorders group, five physical comorbidities showed a significantly higher prevalence in males than females: other neurological disorders (*χ*^2^ = 27.50, *p* < 0.001), liver disease (*χ*^2^ = 69.35, *p* < 0.001), uncomplicated HTN (*χ*^2^ = 48.25, *p* < 0.001), uncomplicated DM (*χ*^2^ = 18.09, *p* < 0.001), and fluid and electrolyte disorders (*χ*^2^ = 21.07, *p* < 0.001). Two physical comorbidities showed a significantly higher prevalence in females than males: iron deficiency anemia (*χ*^2^ = 126.03, *p* < 0.001) and hypothyroidism (*χ*^2^ = 146.83, *p* < 0.001). In the mood disorders group, three physical comorbidities were significantly more prevalent in males than in females: liver disease (*χ*^2^ = 56.18, *p* < 0.001), uncomplicated DM (*χ*^2^ = 4.81, *p* = 0.028), and complicated DM (*χ*^2^ = 4.14, *p* = 0.041). Six physical comorbidities were significantly more prevalent in females than in males: uncomplicated HTN (*χ*^2^ = 7.65, *p* = 0.005), chronic pulmonary disease (*χ*^2^ = 104.92, *p* < 0.001), peptic ulcer disease excluding bleeding (*χ*^2^ = 102.16, *p* < 0.001), peripheral vascular disorders (*χ*^2^ = 13.91, *p* < 0.001), cardiac arrhythmia (*χ*^2^ = 6.81, *p* = 0.009), and hypothyroidism (*χ*^2^ = 298.89, *p* < 0.001).

## 4. Discussion

Individuals with SMI are susceptible to physical comorbidities associated with unhealthy lifestyles, side effects of psychotropic treatments, and poor access to medical care for physical problems [9]. Schizophrenia is a major mental disorder with significant comorbidity and mortality [5]. In this study, 68.6% of patients with schizophrenia-related disorders had at least one physical comorbidity. A study conducted in Iowa in the US using a modified ECM reported that 71.2% of patients with schizophrenia had one or more physical diseases, which was significantly higher than the rate (45.3%) in the general control group [12]. Similar to the previous study, our current results indicate a high degree of physical disease in patients with schizophrenia. In a study examining the medical condition of middle-aged and older homeless people with schizophrenia and depressive disorders, those with schizophrenia had fewer medical visits and recorded medical problems, and were less likely to undergo detailed physical or cancer screenings than those with depression [28]. Likewise, the lack of adequate surveillance and treatment for physical illnesses further explains the high physical comorbidity of schizophrenia, continues to increase physical risk, and eventually contributes to premature death. 

In the mood disorders group, 76.4% of the patients had more than 1 physical disease comorbidity, significantly higher proportion than in the schizophrenia-related disorders group. In a national probability sample of adult household residents (*n* = 16,423) living in the US, two-thirds of middle-aged and elderly adults (≥50 years or older) who met the criteria for major depression reported a diagnosis of comorbid cardiovascular disease [29]. Moreover, a study that examined physical comorbidities in a large sample in the UK reported a significant increase in the self-reported lifetime rate of medical diseases in patients with bipolar disorder (*n* = 1720) compared with the control group (*n* = 1340) [18]. Mood and physical condition are closely related, and evidence of the impact of depression and other mood states on numerous physical illnesses is increasing [30]. Stress stimulation triggers and exacerbates the dysregulation of HPA function, which leads to the physical, cognitive, emotional, and behavioral symptoms of depressive disorder [1]. HPA changes are also associated with peripheral disorders such as metabolic and cardiovascular diseases. Furthermore, certain inflammatory and endocrine pathways seem to interact in both the peripheral and central nervous systems to potentiate the state of psychiatric dysfunction, particularly depressed mood [2]. The increased morbidity and mortality may also be explained by the negative effects of major depressive disorders on health behaviors (e.g., smoking, sedentary lifestyle, poor diet, over-eating), maladaptive effects on adherence to medical regimens, and direct adverse physiological effects [6]. Mental health professionals must understand the importance of mind–body interaction and its effects on mood disorders, and should detect relevant signs and symptoms earlier and refer to the appropriate medical departments to reduce high physical comorbidity rates and improve health outcomes.

In the schizophrenia-related disorders group, other neurological disorders were the most prevalent. This result differed from that of a study reporting that chronic gastritis/gastroesophageal reflux disease was the most prevalent chronic comorbid physical disease among patients with schizophrenia in Korea [7]. This is considered to be due to the differences in tools for measuring physical comorbidities, which differ in their classification system and definitions of physical comorbidities. Thus, when comparing the current status of comorbidities, it is necessary to consider various factors such as the comorbidity measurement system and definition of comorbid diseases. 

Previous studies have also noted the high comorbidity of neurological diseases in schizophrenia [5,12,13,31,32]. In a study by Carney et al. using a modified ECM, other neurological disorders had the second highest odds ratio (OR) compared with the control group (OR = 9.67) [12]. Neurological signs co-occurring with psychopathology are considered a trait feature of schizophrenia, as patients with schizophrenia more often show neurological soft signs, olfactory abnormalities, and epilepsy compared with the general population [13]. These neurological abnormalities may appear several years before the onset of schizophrenia and may occur as extrapyramidal symptoms due to antipsychotic medications [31,32]. Among the neurological disorders, patients with schizophrenia were found to have higher rates of Parkinsonism and epilepsy than hospital control patients with other medical diagnoses [5]. Therefore, in healthcare for patients with schizophrenia, detailed history taking, systematic assessment and examination, and timely referral should be performed to manage nervous system abnormalities or diseases.

In patients with mood disorders, the most prevalent physical comorbidity was liver disease. In a study using Taiwan’s National Institute of Health Database, patients with major depressive disorder (*n* = 766,427) had a significantly higher prevalence (OR = 2.27) and incidence of chronic liver disease than the general population [33]. In the present study, liver disease also showed the second highest prevalence among patients with schizophrenia-related disorders. The use of antipsychotics and antidepressants can affect liver function and cause drug-induced liver injury [34]. Symptoms such as changes in appetite, sleep disturbances, and decreased physical activity in patients with mood disorders also cause metabolic problems [35,36] and can lead to chronic liver disease [33]. Alcohol use disorders often coexist with mood disorders, and alcoholic liver disease is a major health problem [33]. Therefore, it is necessary to assess the risk and onset of liver disease early in patients with mood disorders and refer to the appropriate specialist.

An overall comparison of the two groups of mental disorders revealed that the prevalence of each comorbid physical disease was significantly higher in the mood disorders group. However, many studies reported higher mortality among patients with schizophrenia [5,37]. In particular, the excess all-cause mortality was higher in people with schizophrenia than with bipolar disorder or with unipolar disorder in the Scandinavian study [37]. Our finding is thought to be due to the older age of this group and higher possibility that HPA axis dysfunction induces complex comorbidities and specific inflammatory processes in depression [2,30]. On the other hand, other neurological disorders showed a significantly higher prevalence in those with schizophrenia-related disorders. This is considered related to the use of antipsychotic drugs, which are prescribed mainly for schizophrenia. In particular, typical antipsychotic drugs can further affect the dopamine–acetylcholine balance, leading to secondary Parkinsonism [31]. Therefore, for each mental disorder, a disease-specific treatment approach that considers priorities and higher-risk comorbidities is recommended.

In the prevalence ranking of comorbidities according to sex, male and female patients with schizophrenia-related disorders showed similar patterns, whereas the top-ranking comorbidities in the mood disorders group showed some sex differences. Among patients with mood disorders, liver disease had the highest prevalence in males and third highest in females. Liver disease was also significantly more prevalent in males, in agreement with a study of bipolar disorder patients showing a higher rate of liver diseases/hepatitis in males than females [17]. Chronic liver disease is caused by various factors including genetic factors, alcohol consumption, lifestyle, and metabolic disorders [38,39]. Previous studies on sex-related differences in the factors contributing to alcohol consumption showed that males were more likely to be current, high-frequency, and high-volume drinkers than females [40]. Males are also more likely to drink alcohol in a negative mood compared to females [41]. In bipolar disorder, alcohol use disorder is significantly more common in males than in females [42]. It is necessary to explore factors other than alcohol consumption that cause sex differences in the prevalence pattern of liver disease.

The differences in comorbidity prevalence according to sex were similar in both groups. The prevalence of liver disease and uncomplicated DM was significantly higher in males, and that of hypothyroidism was higher in females. The reason for these differences is found in the prevalence characteristics of the general population. In previous studies of the general adult population in Korea, the prevalence of both liver disease and DM was higher in males than in females [43,44]. Furthermore, females appear to be more knowledgeable about DM than males [45]. In addition, hypothyroidism is more prevalent in females in the general population [46]. Research on sex differences in genetic factors showed that females are more susceptible to developing hypothyroidism [47,48]. 

In summary, we examined the prevalence of physical comorbidities and their differences according to diagnoses and sex among psychiatric inpatients. The results found that the most common physical problems were other neurological disorders in schizophrenia-related disorders and liver disease in mood disorders, and most comorbid physical diseases (except other neurological disorders) were more prevalent in mood disorders than schizophrenia-related disorders. We also found that male and female patients with schizophrenia-related disorders showed similar patterns, whereas the top-ranking comorbidities in the mood disorders group showed some sex differences. In addition, in both diagnostic groups, liver disease and uncomplicated DM were more prevalent in males, whereas hypothyroidism was more prevalent in females. Based on the present findings, patients with mental illness have a variety of comorbid physical health problems. However, mental health professionals who focus on psychological symptoms may overlook physical problems and often skip medical examinations [3]. By focusing only on psychiatric disorders, more complex needs may be missed, resulting in inadequate care [49]. Considering the characteristics of physical comorbidities in psychiatric patients, it is important that mental health professionals become more skilled in assessing the physical problems of people with SMI, and make timely referrals to the relevant medical departments. Health education should include not only mental health issues, but also information on physical diseases with high comorbidity and assessment skills for physical problems. 

This study has some strengths. First, we analyzed national administrative data, rather than data from a single or several clinical settings. These data provide more representative information on the current situation in South Korea. Second, using the ECM, an internationally used comorbidity index, the status of physical comorbidities among psychiatric patients was identified. This will enable a systematic comparison and consideration of our results in future studies using the ECM.

However, some limitations should be considered. First, this study used NHI claims data, which reflect only patients who visited the hospital and received treatment. Even if serious physical health problems exist, patients with mental illness may not be diagnosed during hospital visits. Therefore, the results of this study have limited representation of psychiatric patients with physical disorders. Second, when evaluating comorbid diseases based on these data, the injury or disease code that is advantageous for reimbursement of medical costs according to the Korean health insurance system may be used, and injuries or diseases not related to costs may be omitted. Third, the data used in this study did not provide information on whether patients were born in Korea. If foreigners subscribe to health insurance, they may be included in the NHI data. Despite these limitations, this study analyzed data from all treated patients with schizophrenia-related disorders and mood disorders diagnosed in Korea. Therefore, it provides important information and knowledge on the status of physical comorbidities among psychiatric patients in Korea. This information will contribute to realizing a holistic approach that takes care of both physical and mental health, facilitating the development of effective interventions for the prevention and management of physical comorbidities in people with SMI. 

## 5. Conclusions

Patients with schizophrenia-related disorders and mood disorders exhibited a high prevalence of physical comorbidities. Physical comorbidities in psychiatric patients differed according to diagnoses and sex. Based on information and knowledge on the status of comorbid physical diseases among psychiatric patients, mental health professionals should develop assessment skills for physical health problems and refer to the appropriate specialists to prevent and manage high-risk physical comorbidities. Systematic education should be offered for mental health professionals to provide information on physical comorbidity status and strengthen assessment skills for physical diseases in people with mental illness.

## Figures and Tables

**Table 1:** General characteristics of psychiatric inpatients according to diagnoses (*N* = 48,902).

	Total (*N* = 48,902)	Schizophrenia-Related Disorders (*n* = 26,314)	Mood Disorders (*n* = 22,588)	*T* , *χ*^2^ or *U*	*p*
Age, M ± SD	48.33 ± 15.80	46.54 ± 14.05	50.41 ± 17.39	−27.20	<0.001
Age groups					
19–29	6850 (14.0)	3484 (13.2)	3366 (14.9)	1469.53	<0.001
30–39	8084 (16.6)	4941 (18.8)	3143 (13.9)		
40–49	11,156 (22.8)	6966 (26.5)	4190 (18.6)		
50–59	11,214 (22.9)	6303 (24.0)	4911 (21.7)		
≥60	11,598 (23.7)	4620 (17.5)	6978 (30.9)		
Gender					
Male	22,960 (46.9)	13,292 (50.5)	9668 (42.8)	290.20	<0.001
Female	25,942 (53.1)	13,022 (49.5)	12,920 (57.2)		
Type of insurance					
Medical aid	17,263 (35.3)	11,755 (44.7)	5508 (24.4)	2190.30	<0.001
Insurance	31,639 (64.7)	14,559 (55.3)	17,080 (75.6)		
Length of stay, Median (IQR)	28 (49)	34 (68)	20 (30)	467,025,211.50	<0.001 ^†^
Types of hospitals ^‡^					
Tertiary	4339 (8.9)	1526 (5.8)	2813 (12.5)	2497.02	<0.001
General	10,554 (21.6)	3993 (15.2)	6561 (29.0)		
Hospital	20,275 (41.5)	12,341 (46.9)	7934 (35.1)		
Psychiatric	9604 (19.6)	6134 (23.3)	3470 (15.4)		
Clinic	4130 (8.4)	2320 (8.8)	1810 (8.0)		
Previous psychiatric hospitalization within the last year					
no	31,493 (64.4)	14,322 (54.5)	17,161 (76.0)	2452.62	<0.001
Yes	17,409 (35.6)	11,982 (45.5)	5427 (24.0)		
ECM score for the last year					
0	13,587 (27.8)	8260 (31.4)	5327 (23.6)	369.19	<0.001
≥1	35,315 (72.2)	18,054 (68.6)	17,261 (76.4)		

M = mean; SD = standard deviation; IQR = interquartile range; ECM = Elixhauser Comorbidity Measure; ^†^ *p*-values were calculated using Mann–Whitney *U*; ^‡^ Types of hospitals at hospital level (*n* = 634): tertiary—22 (3.5%), general—122 (19.2%), hospital—224 (35.3%), psychiatric—126 (19.9), clinic—140 (22.1%).

**Table 2:** Prevalence of physical comorbidities according to diagnoses (*N* = 48,902).

Rank	Physical Comorbidities	Schizophrenia-Related Disorders (*n* = 26,314)		Physical Comorbidities	Mood Disorders (*n* = 22,588)	
		*n*	%		*n*	%
1	Other neurological disorders	8501	32.3	Liver disease	7279	32.2
2	Liver disease	6132	23.3	HTN, uncomplicated	6743	29.9
3	Chronic pulmonary disease	5279	20.1	Chronic pulmonary disease	6429	28.5
4	HTN, uncomplicated	4748	18.0	Other neurological disorders	5435	24.1
5	DM, uncomplicated	4150	15.8	Peptic ulcer disease excluding bleeding	4995	22.1
6	Peptic ulcer disease excluding bleeding	3086	11.7	DM, uncomplicated	4818	21.3
7	Fluid and electrolyte disorders	2169	8.2	Peripheral vascular disorders	2650	11.7
8	DM, complicated	1792	6.8	Fluid and electrolyte disorders	2616	11.6
9	Deficiency anemias	1734	6.6	DM, complicated	2453	10.9
10	Peripheral vascular disorders	1407	5.4	Deficiency anemias	1933	8.6

HTN = hypertension; DM = diabetes mellitus.

**Table 3:** Difference in physical comorbidities according to diagnoses (*N* = 48,902).

Physical Comorbidities	Schizophrenia-Related Disorders (*n* = 26,314)		Mood Disorders (*n* = 22,588)		*χ* ^2^	*p*
	*n*	%	*n*	%		
1. CHF	612	2.3	1029	4.6	186.33	<0.001
2. Cardiac arrhythmias	1112	4.2	1641	7.3	211.29	<0.001
3. Valvular disease	43	0.2	117	0.5	46.85	<0.001
4. Pulmonary circulation disorders	31	0.1	76	0.3	26.61	<0.001
5. Peripheral vascular disorders	1407	5.4	2650	11.7	651.30	<0.001
6. HTN						
HTN, uncomplicated	4748	18.0	6743	29.9	942.80	<0.001
HTN, complicated	458	1.7	878	3.9	210.74	<0.001
7. Paralysis	193	0.7	306	1.3	46.44	<0.001
8. Other neurological disorders	8501	32.3	5435	24.1	405.45	<0.001
9. Chronic pulmonary disease	5279	20.1	6429	28.5	471.02	<0.001
10. DM, uncomplicated	4150	15.8	4818	21.3	250.79	<0.001
11. DM, complicated	1792	6.8	2453	10.9	251.45	<0.001
12. Hypothyroidism	1289	4.9	1752	7.8	170.21	<0.001
13. Renal failure	287	1.1	392	1.7	36.90	<0.001
14. Liver disease	6132	23.3	7279	32.2	486.10	<0.001
15. Peptic ulcer disease excluding bleeding	3086	11.7	4995	22.1	950.45	<0.001
16. HIV/AIDS	7	0.03	9	0.04	0.65	0.419
17. Lymphoma	8	0.03	24	0.1	10.69	0.001
18. Metastatic cancer	64	0.2	77	0.3	4.03	0.044
19. Solid tumor without metastasis	586	2.2	924	4.1	141.08	<0.001
20. Rheumatoid arthritis/collagen vascular diseases	595	2.3	1035	4.6	203.19	<0.001
21. Coagulopathy	264	1.0	307	1.4	13.33	<0.001
22. Obesity	44	0.2	53	0.2	2.79	0.094
23. Weight loss	372	1.4	524	2.3	55.48	<0.001
24. Fluid and electrolyte disorders	2169	8.2	2616	11.6	153.47	<0.001
25. Blood loss anemia	61	0.2	82	0.4	7.17	0.007
26. Deficiency anemias	1734	6.6	1933	8.6	67.86	<0.001

CHF = congestive heart failure; HTN = hypertension; DM = diabetes mellitus; HIV/AIDS = human immunodeficiency virus infection and acquired immunodeficiency syndrome.

**Table 4:** Prevalence of physical comorbidities according to diagnoses and sex (*N* = 48,902).

**Diagnosis Group: Schizophrenia-Related Disorders**						
**Rank**	**Physical Comorbidities**	**Male (*n* = 13,292)**		**Physical Comorbidities**	**Female (*n* = 13,022)**	
		***n***	**%**		***n***	**%**
1	Other neurological disorders	4493	33.8	Other neurological disorders	4008	30.8
2	Liver disease	3383	25.5	Liver disease	2749	21.1
3	Chronic pulmonary disease	2619	19.7	Chronic pulmonary disease	2660	20.4
4	HTN, uncomplicated	2615	19.7	HTN, uncomplicated	2133	16.4
5	DM, uncomplicated	2222	16.7	DM, uncomplicated	1928	14.8
6	Peptic ulcer disease excluding bleeding	1517	11.4	Peptic ulcer disease excluding bleeding	1569	12.1
7	Fluid and electrolyte disorders	1198	9.0	Deficiency anemias	1084	8.3
8	DM, comp	939	7.1	Fluid and electrolyte disorders	971	7.5
9	Peripheral vascular disorders	723	5.4	DM, complicated	853	6.6
10	Deficiency anemias	650	4.9	Hypothyroidism	850	6.5
**Diagnosis Group: Mood Disorders**						
**Rank**	**Physical Comorbidities**	**Male (*n* = 9668)**		**Physical Comorbidities**	**Female (*n* = 12,920)**	
		***n***	**%**		***n***	**%**
1	Liver disease	3376	34.9	Chronic pulmonary disease	4021	31.1
2	HTN, uncomplicated	2792	28.9	HTN, uncomplicated	3951	30.6
3	Chronic pulmonary disease	2408	24.9	Liver disease	3903	30.2
4	Other neurological disorders	2343	24.2	Peptic ulcer disease excluding bleeding	3169	24.5
5	DM, uncomplicated	2129	22.0	Other neurological disorders	3092	23.9
6	Peptic ulcer disease excluding bleeding	1826	18.9	DM, uncomplicated	2689	20.8
7	Fluid and electrolyte disorders	1110	11.5	Peripheral vascular disorders	1605	12.4
8	DM, complicated	1097	11.4	Fluid and electrolyte disorders	1506	11.7
9	Peripheral vascular disorders	1045	10.8	DM, complicated	1356	10.5
10	Cardiac Arrhythmias	652	6.7	Hypothyroidism	1346	10.4

**Table 5:** Differences in physical comorbidities according to diagnoses and sex (*N* = 48,902).

**Physical Comorbidities**	**Schizophrenia-Related Disorders (*n* = 26,314)**				***χ*^2^**	***p***
	**Male** **(*n* = 13,292)**		**Female** **(*n* = 13,022)**			
	***n***	**%**	***n***	**%**		
Other neurological disorders	4493	33.8	4008	30.8	27.50	<0.001
Liver disease	3383	25.5	2749	21.1	69.35	<0.001
Chronic pulmonary disease	2619	19.7	2660	20.4	2.14	0.142
HTN, uncomplicated	2615	19.7	2133	16.4	48.25	<0.001
DM, uncomplicated	2222	16.7	1928	14.8	18.09	<0.001
Peptic ulcer disease excluding bleeding	1517	11.4	1569	12.1	2.57	0.108
Fluid and electrolyte disorders	1198	9.0	971	7.5	21.07	<0.001
DM, complicated	939	7.1	853	6.6	2.74	0.098
Peripheral vascular disorders	723	5.4	684	5.3	0.45	0.500
Deficiency anemias	650	4.9	1084	8.3	126.03	<0.001
Hypothyroidism	439	3.3	850	6.5	146.83	<0.001
**Physical Comorbiditiesh**	**Mood Disorders (*n* = 22,588)**				***χ*** **^2^**	***p***
	**Male** **(*n* = 9668)**		**Female** **(*n* = 12,920)**			
	***n***	**%**	***n***	**%**		
Liver disease	3376	34.9	3903	30.2	56.18	<0.001
HTN, uncomplicated	2792	28.9	3951	30.6	7.65	0.005
Chronic pulmonary disease	2408	24.9	4021	31.1	104.92	<0.001
Other neurological disorders	2343	24.2	3092	23.9	0.28	0.598
DM, uncomplicated	2129	22.0	2689	20.8	4.81	0.028
Peptic ulcer disease excluding bleeding	1826	18.9	3169	24.5	102.16	<0.001
Fluid and electrolyte disorders	1110	11.5	1506	11.7	0.17	0.684
DM, complicated	1097	11.4	1356	10.5	4.14	0.041
Peripheral vascular disorders	1045	10.8	1605	12.4	13.91	<0.001
Cardiac arrhythmias	652	6.7	989	7.7	6.81	0.009
Hypothyroidism	406	4.2	1346	10.4	298.89	<0.001

CHF = congestive heart failure; HTN = hypertension; DM = diabetes mellitus; HIV/AIDS = human immunodeficiency virus infection and acquired immunodeficiency syndrome.

## Data Availability

No new data were created or analyzed in this study. Data sharing is not applicable to this article.
